# The diagnostic role of diffusional kurtosis imaging in glioma grading and differentiation of gliomas from other intra-axial brain tumours: a systematic review with critical appraisal and meta-analysis

**DOI:** 10.1007/s00234-020-02425-9

**Published:** 2020-05-04

**Authors:** Gehad Abdalla, Luke Dixon, Eser Sanverdi, Pedro M. Machado, Joey S. W. Kwong, Jasmina Panovska-Griffiths, Antonio Rojas-Garcia, Daisuke Yoneoka, Jelle Veraart, Sofie Van Cauter, Ahmed M. Abdel-Khalek, Magdy Settein, Tarek Yousry, Sotirios Bisdas

**Affiliations:** 1 The Neuroradiological Academic Unit, BRR, UCL IoN, London, UK; 2 Department of Radiology, Mansoura university hospitals, Mansoura, Egypt; 3 Imaging Analysis Centre, Queen Square 8-11, London, WC1N 3BG UK; 4 The National Hospital for Neurology and Neurosurgery UCL Hospitals NHS Trust, London, UK; 5 MRC Centre for Neuromuscular Diseases & Centre for Rheumatology, University College London, London, UK; 6 Jockey Club School of Public Health and Primary Care, Faculty of Medicine, The Chinese University of Hong Kong, Shatin, Hong Kong; 7 NIHR CLAHRC North Thames, Department of Applied Health Research, University College London, London, UK; 8 Department of Global Health and Development, London School of Hygiene and Tropical Medicine, 15-17 Tavistock Place, London, WC1H 9SH UK; 9 Department of Global Health Policy, Graduate School of Medicine, University of Tokyo, Tokyo, Japan; 10 Champalimaud Research, Champalimaud Centre for the Unknown, Lisbon, Portugal; 11 imec-Vision Lab, Department of Physics, University of Antwerp, Antwerp, Belgium; 12 University Hospitals Leuven, Leuven, Belgium

**Keywords:** Magnetic resonance imaging, Diffusion-weighted imaging, Gliomas, Diagnosis

## Abstract

**Purpose:**

We aim to illustrate the diagnostic performance of diffusional kurtosis imaging (DKI) in the diagnosis of gliomas.

**Methods:**

A review protocol was developed according to the (PRISMA-P) checklist, registered in the international prospective register of systematic reviews (PROSPERO) and published. A literature search in 4 databases was performed using the keywords ‘glioma’ and ‘diffusional kurtosis’. After applying a robust inclusion/exclusion criteria, included articles were independently evaluated according to the QUADAS-2 tool and data extraction was done. Reported sensitivities and specificities were used to construct 2 × 2 tables and paired forest plots using the Review Manager (RevMan®) software. A random-effect model was pursued using the hierarchical summary receiver operator characteristics.

**Results:**

A total of 216 hits were retrieved. Considering duplicates and inclusion criteria, 23 articles were eligible for full-text reading. Ultimately, 19 studies were eligible for final inclusion. The quality assessment revealed 9 studies with low risk of bias in the 4 domains. Using a bivariate random-effect model for data synthesis, summary ROC curve showed a pooled area under the curve (AUC) of 0.92 and estimated sensitivity of 0.87 (95% CI 0.78–0.92) in high-/low-grade gliomas’ differentiation. A mean difference in mean kurtosis (MK) value between HGG and LGG of 0.22 (95% CI 0.25–0.19) was illustrated (*p* value = 0.0014) with moderate heterogeneity (*I*^2^ = 73.8%).

**Conclusion:**

DKI shows good diagnostic accuracy in the differentiation of high- and low-grade gliomas further supporting its potential role in clinical practice. Further exploration of DKI in differentiating IDH status and in characterising non-glioma CNS tumours is however needed.

**Electronic supplementary material:**

The online version of this article (10.1007/s00234-020-02425-9) contains supplementary material, which is available to authorized users.

## Introduction

Gliomas are the commonest primary brain tumour and they remain a leading cause of solid cancer-related deaths in the under 40s [1]. Gliomas encompass a heterogeneous broad group of tumours with different cellular origins and variable biological behaviour. Classification of gliomas is therefore essential to guide therapy, anticipate treatment response and predict prognosis. Historically, the old WHO glioma classification was based on a histologic definition of predominant cellular lineage and grade, essentially differentiating gliomas into high-grade more aggressive and low-grade less aggressive [2]. The advent of biomolecular characterisation has led to the identification of key genetic markers which also influence tumour behaviour. The isocitrate dehydrogenase (IDH) gene has received particular recognition and has contributed to a major revision in the latest 2016 World Health Organization classification [3]. Despite these advances, this essential classification remains reliant on invasive tissue sampling. This has considerable risks, including foremost permanent neurological deficit which also greatly precludes repeat sampling to check for high-grade transformation. Tissue sampling is additionally fallible, with biopsies and incomplete resections potentially providing a non-representative sample due to intra-tumoral heterogeneity. Thus, there is a clear need for a non-invasive imaging marker of tumour type, grade and genetic status which would inform management and estimate prognosis.

Diffusion-weighted imaging (DWI) and diffusion tensor imaging (DTI) have a well-established role in the radiological assessment of brain tumours. The utility of these diffusion-based sequences in staging remains suboptimal, however [4]. Both DWI and DTI assume that the diffusion of water molecules involves random, Brownian motion. Following this assumption the probability distribution function (PDF), the chance of a proton diffusing between two points in a given time is thought to follow a Gaussian distribution [5]. The apparent diffusion coefficient (ADC) is based on the standard deviation of this PDF and DTI extends this by deriving the ADC in a direction-dependent manner [5, 6]. Although the Gaussian model in DWI and DTI holds true for pure liquids, it overlooks the in vivo effect of the complex cytoarchitecture of organic tissue formed of various compartments, cell types and intracellular constituents. The true PDF, therefore, exhibits non-Gaussian behaviour and the way this deviates from a Gaussian PDF can be assessed using the dimensionless statistical measure called kurtosis. Diffusion kurtosis imaging (DKI) is a novel extension of DTI and provides the degree of directional, non-Gaussian diffusion, i.e. the diffusion kurtosis tensor [7, 8]. Although the actual physiological basis of DKI remains unclear, the notion is that microstructural variances between gliomas of different grades will result in different DKI parameters, e.g. mean kurtosis (MK), and therefore will potentially provide a more accurate, non-invasive biomarker for glioma staging [5, 9]. Early research is encouraging. Two prior meta-analyses which looked at the diagnostic accuracy of DKI for glioma grading projected a pooled area under the curve (AUC) of 0.94 and 0.96 for MK [10, 11]. We attempted to consolidate the preliminary meta-analyses evidence in the topic through an updated systematic review and meta-analysis. These earlier studies also only analysed the ability of DKI to differentiate glioma grade with no probing of DKIs role in differentiating glioma from other intra-axial tumours. This is an important question as it assesses the real-world applicability of DKI as a non-invasive imaging tool in tumours of an unknown lineage that have not been sampled. To address these issues, our systematic review and meta-analysis will scrutinise two research questions; firstly, we will further assess the diagnostic accuracy of DKI in differentiating low-grade glioma (LGG) from high-grade glioma (HGG) by broadening the inclusion criteria and including recent studies that adhere to the up-to-date WHO 2016 glioma classification and include IDH genotyping. To answer this first question, we will specifically look at the mean difference in MK between HGG and LGG and the overall diagnostic accuracy of DKI. Secondly, for the first time, we will also review the role of DKI in differentiating gliomas from other intra-axial tumours.

## Materials and methods

A comprehensive review protocol was set up according to the Preferred Reporting Items for Systematic Review and Meta-analysis Protocols (PRISMA-P) statement and guidance from the Joanna Briggs Institute Reviewers’ Manual for the systematic review of studies of diagnostic test accuracy [12, 13]. The protocol was registered in the international prospective register of systematic reviews, PROSPERO (registration number: CRD42018099192) and details have been published previously [14]. For the development of this full systematic review, the Preferred Reporting Items for a Systematic Review and Meta-analysis of Diagnostic Test Accuracy Studies (PRISMA-DTA) checklist was used [15]. A detailed PRISMA checklist is available in Online Resource 1.

### Search strategy for identification of studies

A systematic literature search in four databases (PubMed, Medline via Ovid, Scopus and Embase) was conducted on the 12th of July 2018, with the help of librarian KB. The search syntax used the keywords ‘glioma’ and ‘diffusional kurtosis’ as both MeSH (Medical Subject Headings) terms and free text words without language restrictions. Reference lists of the included articles were also searched for keywords. A detailed search strategy is given in Online Resource 2.

### Inclusion and exclusion criteria

Studies were eligible for inclusion if they assessed DKI in (i) the diagnosis of primary or recurrent glioma using either the WHO 2007 or the WHO 2016 classifications; or (ii) the differentiation of gliomas in comparison with other brain tumours. Exclusion criteria comprised paediatric age groups, non-original research articles (review, commentaries, erratum, books, editorial and conference abstracts), animal studies, non-imaging studies, non-MRI studies, non-kurtosis MRI studies, non-neoplastic conditions, non-glial tumours only, non-cerebral tumours and studies written in languages other than English, French or German. Non-relevant studies were excluded following a reading of eligible articles in full text.

### Methodological quality (risk of bias) assessment

Risk of bias and applicability concerns were assessed independently by two authors (GA and SES) using the QUADAS-2 tool (revised tool of quality assessment of diagnostic accuracy studies) [16]. Any disagreements were resolved in consensus. If an agreement could not be reached, a third reviewer was available as an adjudicator (PMM). Quality assessment was performed twice separately for each of the two review questions. Risk of bias was assessed in four domains: patient selection, index test, reference standard, and flow and timing. Applicability concerns were assessed in three domains: patient selection, index test, and reference standard. The risk of each domain was judged to be high, low, or unclear. Risk of bias across studies (i.e. ‘publication bias’) was not assessed as there remains no universally accepted method and the number of studies was small [17]. For the risk of bias domains, certain adaptations were outlined: (1) In patient selection prospective, studies were deemed low risk whilst retrospective studies were judged high risk. (2) For index test, studies were judged low risk if the radiologist was blinded to the reference standard during image analysis versus high risk if unblinded. (3) Reference standard was defined as histopathological assessment. (4) Inflow and timing unclear risk was recorded if the interval between the index test and reference standard was not given or if patients were removed from the study without defined reason.

### Data extraction

Data extraction was conducted by GA and SES using pre-designed standardized sheets. Extracted data included the name of the first author, publication year, study type, patient population demographics, acquisition techniques, processing and post-processing software, reference standard, WHO classification scheme and diagnostic test accuracy results (true and false positive and negative values).

### Overlapping datasets

Studies that include the same authors will be checked for overlapping patient cohorts through direct contact with the study’s author/s. If there are overlapping cohorts across different studies, then the most recently published study from that group will be included in the analysis.

### Data synthesis and analyses

Mean and standard deviation of mean kurtosis (MK) value were used to describe the mean differences between low-grade glioma (LGG) and high-grade glioma (HGG) groups with a random-effects meta-analysis model using the restricted maximum likelihood method. To analyse the heterogeneity and the robustness of the results, we performed subgroup analysis by stratifying the included studies according to the type of technique such as the time of repetition (TR) value, number of *b* values (i.e. the degree of diffusion weighting), maximum *b* value and diffusion direction. As a measure of the degree of heterogeneity between the studies, we used the *Q* and *I*^2^ statistics.

For analysis of diagnostic test accuracy (DTA), a bivariate random-effects meta-analysis using the restricted maximum likelihood method was used to calculate the summary receiver operating characteristic (ROC) curve and its area under the curve (AUC). Coupled plots showing points of sensitivity and false-positive rate with their 95% confidence intervals (CI) were also calculated. Bivariate meta-regression was implemented to assess study level characteristics: time to echo (TE), TR, max *b* value, number of *b* values and number of diffusion directions. Statistical analysis was performed with R (version 3.5.1).

## Results

### Search results and included studies

**Fig. 1:**
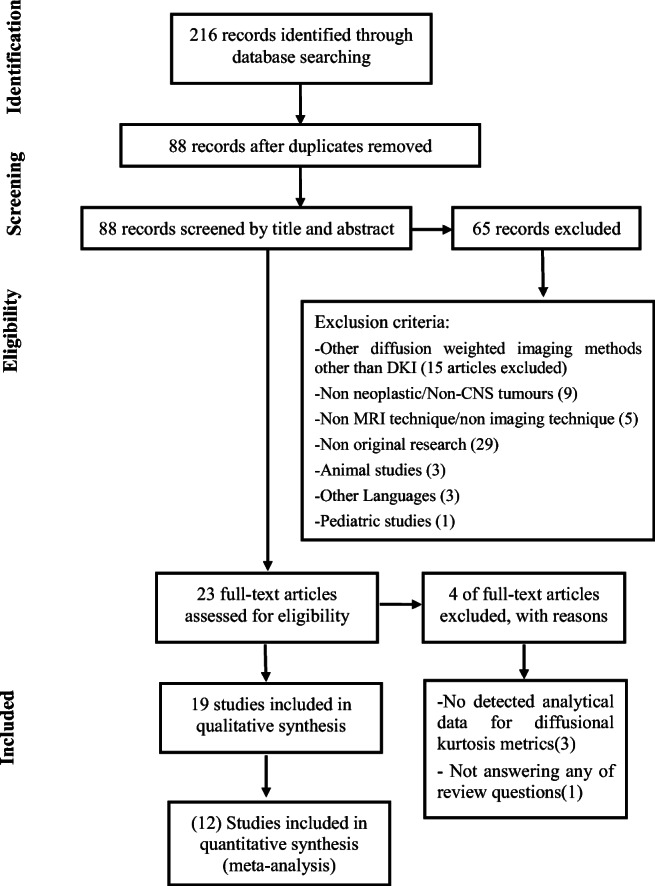
Flowchart for selection of the included in the meta-analysis studies

A systematic search of the four databases led to the identification of 216 studies. After checking for duplicates, 88 studies remained. Of these, 65 were excluded based on the pre-defined inclusion and exclusion criteria by reading titles and abstracts. The remaining 23 articles were selected for full-text evaluation. Following a detailed assessment, 3 studies were excluded owing to lack of analytical data for DKI and 1 study was excluded because it did not answer any of the two pre-defined review questions. Finally, 19 studies were deemed to be eligible for inclusion in the systematic review [9, 18–35]. For the primary question, investigating the role of DKI in glioma grading 17 studies was selected [9, 20–35]. For the secondary question, assessing the technique’s potential in differentiating glioma from other intra-axial tumours the remaining 2 studies was selected [18, 19]. The results of the selection process are presented in Fig. 1. The studies with their characteristics in the areas of histologic types of glioma, details of DKI acquisition technique (e.g. TR/TE, *b* values and diffusion encoding directions), DKI processing and post-processing (e.g. software used and extracted parameters) are illustrated in Table 1.

**Table 1:** Summary of findings for the studies included in the systematic literature review

Authors, publication year	Study type	Study population					DKI acquisition technique					DKI processing				Reference Standard
		Patients’ no.	Mean age (year)	Histologic types, (*n*)	Molecular types, (*n*)	Recurrent cases (*n*)	Machine, strength	DKI:TR/TE (ms)	*b* value no.	Max *b* value(s/mm^2^)	Diffusion directions (*n*)	ROI, sequence used	Normalisation	DKI parameter/s	Time interval (days)	
Hempel et al. 2017	Prospective	60	51.4 ± 14.6	AS II(17), AS III(15), AS IV(12), OD II(11), OD III(5)	IDH mut AS (22), IDH wt GBM (22), 1p/19q OD (16)	None	Siemens, 3T	5900/95	6	2500	30	Whole solid tumour, FLAIR	cNAWM	MK	14	Histologic and immunohistochemistry (IDH, ATRX) examination (WHO 2016)
Hempel et al. 2017	Prospective	77	51.3 ± 15.3	AS II(17), AS III(19), AS IV(19), OD II(15), OD III(7)	IDH mut AS (25), IDH wt GBM(30), 1p/19q OD(22)	None	Siemens, 3T	5900/95	6	2500	30	Whole solid tumour, FLAIR	None	MK	14	Histologic and immunohistochemistry examination (WHO 2016)
Hempel et al. 2017	Prospective Data	77	51.3 ± 15.3	AS II(17), AS III(19), AS IV(19), OD II(15), OD III(7)	IDH mutant AS (25), IDH wild type GBM (30), 1p/19q LOH OD (22)	None	Siemens, 3 T	5900/95	6	2500	30	Whole solid tumour, FLAIR	NA	Perfusion biased and corrected MK	14	Histopathology and immunohistochemistry (WHO 2016)
Maximov et al. 2017	Retrospective	24	Range (18–59)	AS II(8), AS III(8), IV(8)	None	None	GE, 3 T	10,000/103.4	3	2500	60	Whole solid tumour, NA	cNAWM	MK, Kr, Ka	7–14	Histopathologic examination (WHO 2007)
Qi et al. 2017	Retrospective	163	40.5 ± 11.5	II(63), III(48), IV(52)	None	None	Siemens, 3 T	3600/76	3	2000	32	Whole solid tumour, T2WI	cNAWM	MK, Kr, Ka	21	Histopathologic examination (WHO 2007)
Qi et al., 2017	Retrospective	39	LGG(40.08), HGG(54.35)	HGG(26), LGG(13)	None	None	Siemens, 3 T	6000/98	3	2000	30	Whole solid tumour, diffusion maps	NA	MK,KFA	NA	Histopathologic examination (WHO 2007)
Delgado et al. 2017	Prospective	35	48 ± 15	AS II(10), AS III(8), OD II(13), OD III(4)	None	None	Philips, 3 T	5400/76	5	2750	15	Whole, centre, peripheral tumour, FLAIR	None	Mk, Kr, Ka	NA	Histopathologic examination (WHO 2007)
Hempel et al. 2016	Prospective	50	49.9 ± 14.2	II(25), III(15), IV(10)	IDH mut (19), IDH wt (15)	16	Siemens, 3T	5900/95	6	2500	30	Whole solid tumour, FLAIR, T2WI	cNAWM	MK	14	Histologic and immunohistochemistry (WHO 2016)
Li et al. 2016	Retrospective	37	47	LGG(16), HGG(21)	None	None	Siemens, 3 T	3000/109	6	2500	30	Centre, peripheral tumour, T2,T1 + C	cNAWM	MK	7	Histopathologic findings (WHO 2007 & KI 67, TVA, TCD)
Tan et al. 2016	Retrospective	60	49.6 HGA = 55.6, LGA = 40.2	HGA(35), LGA(25)	None	None	GE, 3 T	6500/11	3	2000	30	Whole solid tumour, FLAIR, T2, ADC	cNAWM	MK, Kr, Ka	NA	Histopathology (2007) and aquaporin 4 expression
Raja et al. 2016	Retrospective	53	Range = 20–67	II(19), III(20), IV(14)	None	None	Philips, 3 T	4000/104	3	2500	15	Whole solid tumour, T2WI	NA	Mk,Kr, Ka, FAK,	NA	Histopathologic (WHO 2007)
Jiang et al. 2015	Prospective	74	41.36 ± 14.01	I(3), II(31), III(19), IV(21)	None	7	GE, 3 T	6500/85	4	2500	25	Whole solid tumour, T1 + C	cNAWM	MK, Kr, Ka	25	Histopathologic (WHO 2007) including Ki 67
Bai et al. 2015	Prospective	62	46	AS II(15), OD II(8), OAS II(5), OD III(2), OAS III(5), AS III(8), IV(19)	None	None	GE, 3 T	7000/80	6	2500	25	Whole solid tumour, ADC	NA	MK	10	Histopathologic ex WHO 2007
Tietze et al. 2015	Retrospective	34	NA	HGG(22), LGG(12)	None	2	Siemens, 3 T	10,300/100	3	2500	9	Centre, peripheral tumour, FLAIR, T1 + C	cNAWM	MK’(fast MK)	NA	Histopathologic examination WHO-2007
Cauter et al. 2014	Prospective	35 + 19 (validation)	Median age: 55	HGG(21), LGG(14)	None	None	Philips, 3 T	3200/90	3	2800	25, 40, 75	Whole solid tumour, T2WI	NA	MK	19	Histopathologic examination (WHO 2007)
Cauter et al. 2012	Prospective	27	54,HGG = 61, LGG = 42	HGG(17), LGG(11)	None	None	Philips, 3 T	3200/90	3	2800	25,40,75	Whole solid tumour, T2WI	cNAWM, PLIC	MK, Ka, Kr	28	Histopathologic ex WHO-2007, (4 patients depended on PET)
Raab et al. 2010	Prospective	34	56	AS II(5), AS III(13), IV(16)	None	None	Siemens, 3 T	2300/109	6	2500	30	Whole solid tumour, T2WI	cNAWM	MK	21	Histopathologic examination WHO-2007
Pang et al. 2016	Prospective	31	49.2	HGG(20), B lymphoma (11)	None	None	GE, 3 T	5000/90	3	2500	5, 25, 25	Whole solid tumour, FLAIR, T1 + C	cNAWM	MK, Ka, Kr	6	Histopathology examination (WHO 2007)
Tan et al. 2015	Retrospective	51	AS: 56.6, metastasis: 60.1	HGA(31), solitary metastasis (20)	None	None	GE, 3 T	6500/115	3	2000	30	Whole solid tumour, T1 + C	cNAWM	MK, Kr, Ka	NA	Histopathologic examination WHO 2007

*LGG*, low-grade glioma; *HGG*, high-grade glioma; *HGA*, high-grade astrocytoma; LGA, low-grade astrocytoma; *AS*, astrocytoma; *OD*, oligodendroglioma; *IDH mut AS*, isocitrate dehydrogenase mutant astrocytoma; IDH wt GBM, isocitrate dehydrogenase wild-type glioblastoma; *LOH*, loss of heterogeneity; *ms*, milliseconds; *ROI*, region of interest; *T1 + C*, T1-weighed images with contrast; *ADC*, apparent diffusion coefficient; *cNAWM*, contralateral normal appearing white matter; *MK*, mean kurtosis; *Kr*, radial kurtosis; *Ka*, axial kurtosis; *KFA*, kurtosis fractional anisotropy

### Quality assessment

#### First question: role of DKI in glioma grading

**Fig. 2:**
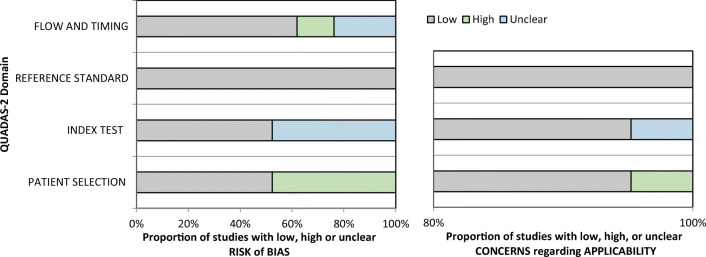
Quality assessment of included diagnostic accuracy studies

Seventeen studies addressed the first question and the results of quality assessment are summarized in Fig. 2 and Table 2 [9, 20–35]. For risk of bias, the quality was variable across each domain. In the patient selection domain, 10 were judged to be at low risk [9, 20–22, 26, 30–35] and 7 were considered high risk [23–25, 27–29]. In the index test domain, 9 studies were considered low risk whilst the remaining 8 were unclear in risk. For the reference standard domain, all studies were deemed low risk. Regarding flow and timing, 11 studies had low risk, 2 studies had high risk and 4 studies had unclear risk. For applicability, all studies across all domains were considered low risk.

**Table 2:**
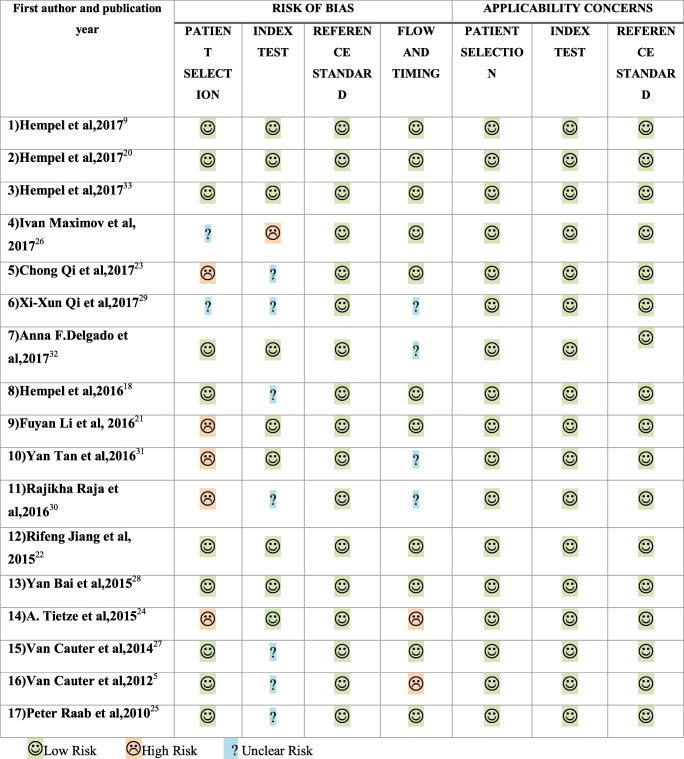
Detailed quality assessment of included diagnostic accuracy studies considering grading of gliomas

#### Second question: role of DKI in differentiating gliomas from other brain tumours

The two included studies addressing this question encountered low risk of applicability and reference standard bias risk [18, 19]. However, the risk of bias was high in patient selection and unclear in flow and timing in one study [19]. Whilst the other study had an unclear risk of bias regarding the index test as blinding of the neuro-radiologist to pathology was not mentioned [18].

### Overlapping datasets

Six studies included in the systematic review were from the same study group and used overlapping patient cohorts and datasets. This was confirmed after contacting these studies authors. In consensus, our team decided to include in the meta-analysis the most recently published work to avoid repetition bias.

### Meta-analyses

For the first question, assessing the role of DKI in differentiating HGGs from LGGs 12 studies out of the 19 selected studies was included in the meta-analysis after considering overlapping datasets [20–31], in addition to another excluded study which evaluated the role of DKI in low-grade glioma only [32]. As previously outlined for the first question, two separate meta-analyses were performed: one assessing the mean difference of mean kurtosis (MK) between high-grade gliomas (HGGs) and low-grade gliomas (LGGs) and the other looking at the overall diagnostic accuracy of DKI. A separate meta-analysis for the second question was not feasible due to the limited number of studies and associated data.

#### Meta-analysis of mean difference

**Fig. 3:**
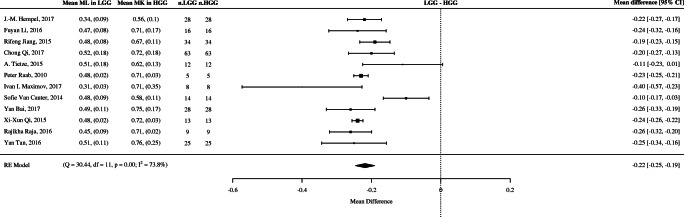
Forest plot of mean difference in MK between LGG and HGG with a random-effects meta-analysis model

All 12 selected studies provided sufficient data to assess mean kurtosis (MK) difference [20–31]. The random-effect model showed a significant difference in MK (pooled mean value of 0.22 (95% CI 0.25–0.19) and *p* value = 0.0014) between HGGs and LGGs. Forest plots of mean difference in MK between LGG and HGG are shown in Fig. 3. Although a moderate degree [33] of heterogeneity was detected between the studies (*I*^2^ = 73.8%), the robustness of our results was verified by sensitivity analysis of multiple study characteristics which showed no significant statistical difference between these features (See Online Resourse 3). Owing to the degree of heterogeneity, it was not possible to define an MK cutoff value for differentiating HGGs from LGGs. Allowing for this, based on the range of cutoff values used across each study, the optimal cutoff value appears to lie between 0.5 and 0.6.

#### Meta-analysis of diagnostic test accuracy

**Fig. 4:**
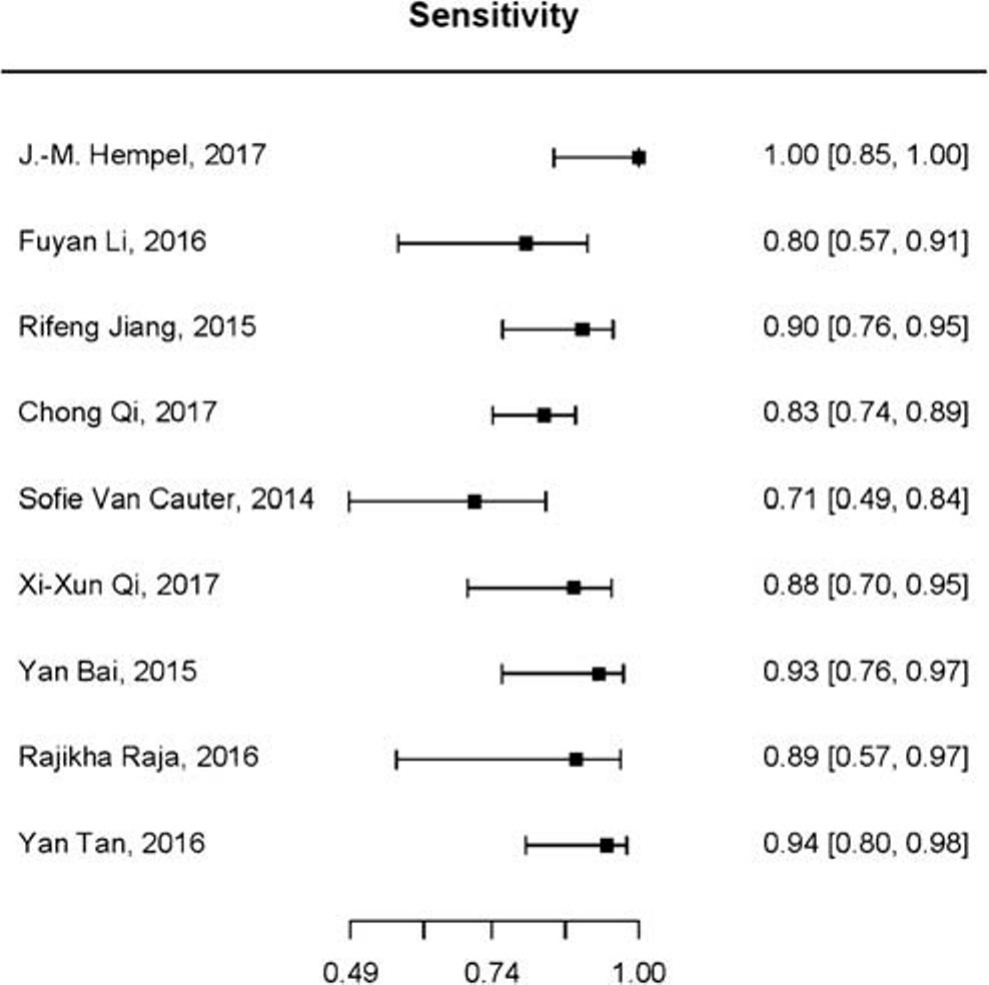
Pooled sensitivity of diffusion kurtosis imaging in the grading of CNS gliomas

**Fig. 5:**
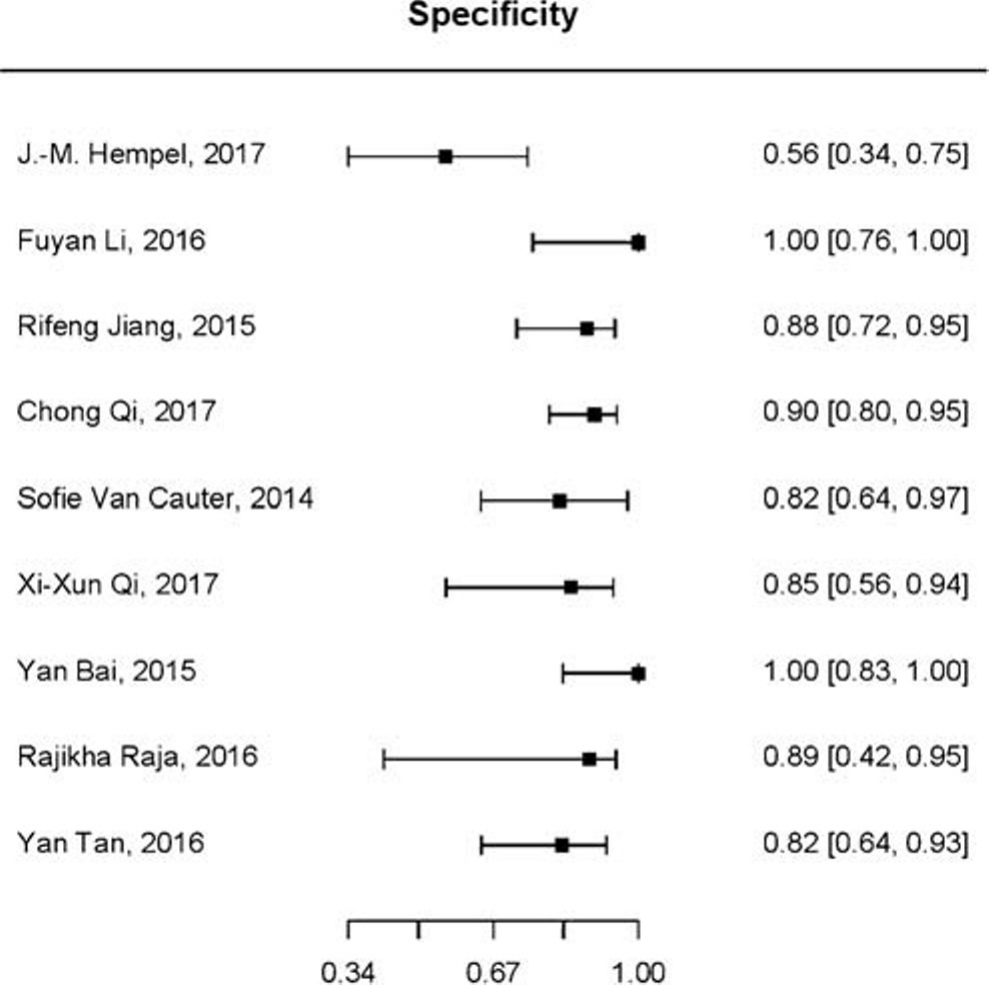
Pooled specificity of diffusion kurtosis imaging in the grading of CNS gliomas

**Fig. 6:**
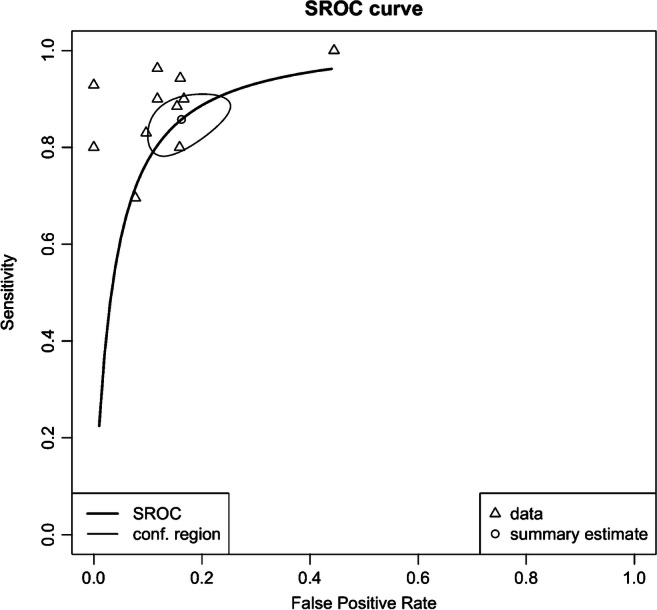
Summary receiver operating characteristic curve

For diagnostic test accuracy (DTA) of DKI in differentiating HGGs from LGGs, 9 of the 12 selected studies were eligible for a bivariate random-effect meta-analysis [20–25, 29–31]. The pooled sensitivity was 0.87 (95% CI 0.78–0.92) and specificity was 0.85 (95% CI 0.76–0.91). Forest plots of sensitivity and specificity of studies are shown in Figs. 4 and 5, respectively. The summary receiver operating characteristic (ROC) curve is shown in Fig. 6 with an area under the curve (AUC) of 0.92. DTA analysis revealed that the DKI false-positive rate was 0.15 (95% CI 0.09–0.24). Bivariate meta-regression model showed study level characteristics such as TE, TR, max *b* value, no. of *b* values and number of diffusion direction had no significant effect on diagnostic accuracy (*p* = 0.155 to *p* = 0.893).

## Discussion

To date, there have been only two published meta-analyses which have looked at the diagnostic test accuracy (DTA) of DKI in glioma discrimination and both demonstrated very promising initial results [10, 11]. Our systematic review and meta-analysis extend this further by increasing the number of electronic databases, removing language restrictions and broadening the eligibility criteria. Importantly regarding DTA of DKI in addition to studies, which follow the WHO 2007 classification, we also included studies using the current WHO 2016 classification, the latter the IDH genotype. Our systematic review included a total of 19 studies, and for looking at the role of DKI in glioma stratification, two meta-analyses were performed. For mean difference analysis of mean kurtosis (MK) across 12 studies, we demonstrated a statistically significant mean difference in MK between high-grade gliomas and low-grade gliomas of 0.22 (95% CI 0.19–0.25). These results are comparable with the Delgado et al., where across 10 studies, they found a significant MK mean difference between high- and low-grade gliomas of 0.17 (95% CI 0.11–0.22) [10]. Our second meta-analysis included 9 studies, assessing the overall diagnostic test accuracy of DKI in grading gliomas, further confirmed its high diagnostic potential with an 87% sensitivity, 85% specificity and 0.92 pooled area under the curve. This is similar to the Delgado group findings which were across a smaller group of 5 studies, which produced a pooled sensitivity and specificity of 85% and 92% respectively and a pooled area under the curve of 0.94.

Looking at heterogeneity, the Delgado et al. subgroup analysis found the type of astrocytoma, the maximum *b* value and repetition time as significant study level characteristics moderating the diagnostic performance of DKI. Our larger study similarly showed moderate heterogeneity in study scanning parameters (such as TE, TR, max *b* value, number of diffusion directions). However, across a wider range of studies and by using a bivariate meta-regression model, we found no significant impact of the different study technical characteristics on the diagnostic accuracy. This finding is particularly encouraging as it suggests generalisable clinical utility. Nevertheless, optimization and standardisation of these parameters and post-processing techniques are still required to enable multi-centre quantitative studies, a key concept in generating higher-level evidence. It was not possible to determine a definite MK cutoff value for differentiating HGGs from LGGs. Despite this limitation based on the range of thresholds used in each separate study, we can extrapolate that the optimal cutoff value for MK is placed between 0.5 and 0.6.

Of the 19 studies in the systematic review, 4 studies looked into DKIs role in stratifying IDH mutation status as per the 2016 WHO classification [9, 20, 34, 35]. These studies reported significantly different MK values between IDH wild type and mutant suggesting its potential role as a surrogate marker for IDH phenotyping. Unfortunately, all 4 studies were from the same institute and based on an overlapping dataset preventing a subgroup meta-analysis [9, 20, 34, 35]. Recently, however, after the date this systematic review search was performed, two studies have been published comparing the diagnostic performance of DKI and DTI in predicting the IDH mutation status in gliomas [36, 37]. Both groups considered MK and mean diffusivity (MD) as the main DKI and DTI parameters respectively and concluded that MK can identify IDH mutation status with higher diagnostic value than MD [36, 37].

For the second question, looking at the role of DKI in differentiating glioma from non-gliomatous CNS tumours, our literature search only identified two studies which did not allow us to obtain any conclusive results [18, 19]. These studies showed however encouraging results that need to be reproduced in the future. Yan Tan et al. analysed MK values in solid tumour parts and the periphery of high-grade astrocytomas (HGAs) and solitary metastatic lesions, concluding that MK values differed significantly in the periphery between the two entities. MK values were also more sensitive than diffusion tensor imaging (DTI) metrics [19]. Using DKI, Pang et al. aimed to differentiate between HGGs and primary CNS lymphomas (PCNSLs) [18]. They reported significantly higher MK in PCNSLs than HGG, which could perhaps be explained by the hypercellular nature of lymphomas microenvironment.

Our study has a few possible limitations. Several of the studies are of limited sample size; none of the included studies reported individual patient data and it is not possible to account for differences in post-processing techniques like tumour ROI. Finally, the bivariate meta-regression analysis lacked a multivariate assessment.

## Conclusion

Our work further confirms that DKI has a very good diagnostic performance in stratifying high- and low-grade gliomas. The consistent accuracy across different studies with varied acquisition and post-processing techniques importantly also implies that DKI is a technique that may be generalisable and clinically useful across different institutions and populations. Optimisation and standardisation of DKI techniques are still needed however to ensure consistency and parity. We also show its potential role as a surrogate marker for IDH phenotyping, although this requires further investigation. Finally, this study highlights the need to further explore the role of DKI in characterising non-glioma tumours.

## Electronic supplementary material

ESM 1 (PDF 392 kb)

ESM 2 (PDF 493 kb)

ESM 3 (PDF 255 kb)

ESM 4 (PDF 384 kb)

ESM 5 (PDF 227 kb)

ESM 6 (PDF 405 kb)

ESM 7 (PDF 344 kb)

ESM 8 (PDF 324 kb)

ESM 9 (PDF 282 kb)

ESM 10 (PDF 344 kb)

## Abbreviations

ADC: Apparent diffusion coefficient
AUC: Area under the curve
CI: Confidence interval
CNS: Central nervous system
DKI: Diffusional kurtosis imaging
DTA: Diagnostic test accuracy
DTI: Diffusion tensor imaging
DWI: Diffusion-weighted imaging
HGAs: High-grade astrocytomas
HGG: High-grade glioma
IDH: Isocitrate dehydrogenase
LGG: Low-grade glioma
MD: Mean diffusivity
MeSH: Medical Subject Headings
MK: Mean kurtosis
PCNSLs: Primary CNS lymphomas
PDF: Probability distribution function
PRISMA-DTA: Preferred Reporting Items for a Systematic Review and Meta-analysis of Diagnostic Test Accuracy Studies
PRISMA-P: Preferred Reporting Items for Systematic Review and Meta-analysis Protocols
PROSPERO: Prospective register of systematic reviews
QUADAS-2 tool: Revised tool of quality assessment of diagnostic accuracy studies
RevMan®: Review Manager
ROC: Receiver operator characteristics
ROI: Region of interest
TE: Time of echo
TR: Time of repetition
WHO: World Health Organization

## References

[1] Ostrom QT, Gittleman H, Truitt G, Boscia A, Kruchko C, Barnholtz-Sloan JS (2018). CBTRUS statistical report: primary brain and other central nervous system tumors diagnosed in the United States in 2011–2015. Neuro-Oncology.

[2] Louis DN, Ohgaki H, Wiestler OD, Cavenee WK, Burger PC, Jouvet A, Scheithauer BW, Kleihues P (2007). The 2007 WHO classification of tumours of the central nervous system. Acta Neuropathol.

[3] Louis DN, Perry A, Reifenberger G, von Deimling A, Figarella-Branger D, Cavenee WK, Ohgaki H, Wiestler OD, Kleihues P, Ellison DW (2016). The 2016 World Health Organization classification of tumors of the central nervous system: a summary. Acta Neuropathol.

[4] Usinskiene J, Ulyte A, Bjørnerud A, Venius J, Katsaros VK, Rynkeviciene R, Letautiene S, Norkus D, Suziedelis K, Rocka S, Usinskas A, Aleknavicius E (2016). Optimal differentiation of high- and low-grade glioma and metastasis: a meta-analysis of perfusion, diffusion, and spectroscopy metrics. Neuroradiology.

[5] Van Cauter S, Veraart J, Sijbers J, Peeters RR, Himmelreich U, De Keyzer F (2012). Gliomas: diffusion kurtosis MR imaging in grading. Radiology.

[6] Basser PJ, Mattiello J, LeBihan D (1994). MR diffusion tensor spectroscopy and imaging. Biophys J.

[7] Jensen JH, Helpern JA, Ramani A, Lu H, Kaczynski K (2005). Diffusional kurtosis imaging: the quantification of non-Gaussian water diffusion by means of magnetic resonance imaging. Magn Reson Med.

[8] Lu H, Jensen JH, Ramani A, Helpern JA (2006). Three-dimensional characterization of non-Gaussian water diffusion in humans using diffusion kurtosis imaging. NMR Biomed.

[9] Hempel J-M, Bisdas S, Schittenhelm J, Brendle C, Bender B, Wassmann H, Skardelly M, Tabatabai G, Vega SC, Ernemann U, Klose U (2017). In vivo molecular profiling of human glioma using diffusion kurtosis imaging. J Neuro-Oncol.

[10] Falk Delgado AA, Nilsson M, van Westen D, Falk Delgado AA (2017) Glioma grade discrimination with MR diffusion kurtosis imaging: a meta-analysis of diagnostic accuracy. *Radiology*:171315. 10.1148/radiol.2017171315 10.1148/radiol.2017171315 29206593

[11] Huang R, Chen Y, Li W, Zhang X (2018) An evidence-based approach to assess the accuracy of diffusion kurtosis imaging in characterization of gliomas. 10.1097/MD.0000000000013068 10.1097/MD.0000000000013068 PMC6221635 30383687

[12] Shamseer L, Moher D, Clarke M, Ghersi D, Liberati A, Petticrew M, et al. Preferred reporting items for systematic review and meta-analysis protocols (PRISMA-P) 2015: elaboration and explanation OPEN ACCESS. 10.1136/bmj.g7647 10.1136/bmj.g7647 25555855

[13] Campbell J, Kulgar M, Carmody D, Hakonsen S, Jadotte Y, White S et al (2017) Chapter 9: Diagnostic test accuracy systematic reviews. In: Aromataris E, Munn Z (Editors). Joanna Briggs Institute reviewer’s manual. *The Joanna Briggs Institute, 2017*

[14] Abdalla G, Sanverdi E, Machado PM, Kwong JSW, Panovska-Griffiths J, Rojas-Garcia A, Yoneoka D, Yousry T, Bisdas S (2018). Role of diffusional kurtosis imaging in grading of brain gliomas: a protocol for systematic review and meta-analysis. BMJ Open.

[15] McInnes MDF, Moher D, Thombs BD, McGrath TA, Bossuyt PM, Clifford T (2018). Preferred reporting items for a systematic review and meta-analysis of diagnostic test accuracy studies. JAMA.

[16] Whiting PF, Rutjes AWS, Westwood ME, Mallett S, Deeks JJ, Reitsma JB (2011). QUADAS-2: a revised tool for the quality assessment of diagnostic accuracy studies. Ann Intern Med.

[17] McInnes MDF, Bossuyt PMM (2015). Pitfalls of systematic reviews and meta-analyses in imaging research. Radiology.

[18] Pang H, Ren Y, Dang X, Feng X, Yao Z, Wu J, Yao C, di N, Ghinda DC, Zhang Y (2016). Diffusional kurtosis imaging for differentiating between high-grade glioma and primary central nervous system lymphoma. J Magn Reson Imaging.

[19] Tan Y, Wang X-C, Zhang H, Wang J, Qin J-B, Wu X-F (2015). Differentiation of high-grade-astrocytomas from solitary-brain-metastases: comparing diffusion kurtosis imaging and diffusion tensor imaging. Eur J Radiol.

[20] Hempel J-M, Schittenhelm J, Bisdas S, Brendle C, Bender B, Bier G et al (2017) In vivo assessment of tumor heterogeneity in WHO 2016 glioma grades using diffusion kurtosis imaging: diagnostic performance and improvement of feasibility in routine clinical practice. J Neuroradiol. 10.1016/j.neurad.2017.07.005 10.1016/j.neurad.2017.07.005 28865921

[21] Li F, Shi W, Wang D, Xu Y, Li H, He J, Zeng Q (2016). Evaluation of histopathological changes in the microstructure at the center and periphery of glioma tumors using diffusional kurtosis imaging. Clin Neurol Neurosurg.

[22] Raja R, Sinha N, Saini J, Mahadevan A, Rao KN, Swaminathan A (2016). Assessment of tissue heterogeneity using diffusion tensor and diffusion kurtosis imaging for grading gliomas. Neuroradiology.

[23] Tan Y, Zhang H, Zhao R-F, Wang X-C, Qin J-B, Wu X-F (2016). Comparison of the values of MRI diffusion kurtosis imaging and diffusion tensor imaging in cerebral astrocytoma grading and their association with aquaporin-4. Neurol India.

[24] Jiang R, Jiang J, Zhao L, Zhang JJ, Zhang S, Yao Y (2015). Diffusion kurtosis imaging can efficiently assess the glioma grade and cellular proliferation. Oncotarget.

[25] Qi C, Yang S, Meng L, Chen H, Li Z, Wang S, Jiang T, Li S (2017). Evaluation of cerebral glioma using 3T diffusion kurtosis tensor imaging and the relationship between diffusion kurtosis metrics and tumor cellularity. J Int Med Res.

[26] Tietze A, Hansen MB, Østergaard L, Jespersen SN, Sangill R, Lund TE (2015). Mean diffusional kurtosis in patients with glioma: initial results with a fast imaging method in a clinical setting. Am J Neuroradiol.

[27] Raab P, Hattingen E, Franz K, Zanella FE, Lanfermann H (2010). Cerebral gliomas: diffusional kurtosis imaging analysis of microstructural differences. Radiology.

[28] Maximov II, Tonoyan AS, Pronin IN (2017). Differentiation of glioma malignancy grade using diffusion MRI. Phys Med.

[29] Van Cauter S, De Keyzer F, Sima DM, Sava AC, D’Arco F, Veraart J (2014). Integrating diffusion kurtosis imaging, dynamic susceptibility-weighted contrast-enhanced MRI, and short echo time chemical shift imaging for grading gliomas. Neuro-Oncology.

[30] Bai Y, Lin Y, Tian J, Shi D, Cheng J, Haacke EM (2016). Grading of gliomas by using monoexponential, biexponential, and stretched exponential diffusion-weighted MR imaging and diffusion kurtosis MR imaging. Radiology.

[31] Qi X-X, Shi D-F, Ren S-X, Zhang S-Y, Li L, Li Q-C et al (2017) Histogram analysis of diffusion kurtosis imaging derived maps may distinguish between low and high grade gliomas before surgery. European Radiology:1–8. 10.1007/s00330-017-5108-1 10.1007/s00330-017-5108-1 29143940

[32] Delgado AF, Fahlström M, Nilsson M, Berntsson SG, Zetterling M, Libard S (2017). Diffusion kurtosis imaging of gliomas grades II and III - a study of perilesional tumor infiltration, tumor grades and subtypes at clinical presentation. Radiol Oncol.

[33] Bossuyt P, Davenport C, Deeks J, Hyde C, Leeflang M, Scholten R Cochrane handbook for systematic reviews of diagnostic test accuracy Chapter 11 Interpreting results and drawing conclusions

[34] Hempel J-M, Schittenhelm J, Brendle C, Bender B, Bier G, Skardelly M (2017). Histogram analysis of diffusion kurtosis imaging estimates for in vivo assessment of 2016 WHO glioma grades: a cross-sectional observational study. Eur J Radiol.

[35] Hempel J-M, Schittenhelm J, Brendle C, Bender B, Bier G, Skardelly M et al (2017) Effect of perfusion on diffusion kurtosis imaging estimates for in vivo assessment of integrated 2016 WHO glioma grades: a cross-sectional observational study. Clin Neuroradiol:1–11. 10.1007/s00062-017-0606-8 10.1007/s00062-017-0606-8 28702832

[36] Tan Y, Zhang H, Wang X, Qin J, Wang L, Yang G, Yan H (2019). Comparing the value of DKI and DTI in detecting isocitrate dehydrogenase genotype of astrocytomas. Clin Radiol.

[37] Zhao J, Wang Y, Li X, Hu M, Li Z, Song Y, Wang JY, Tian YS, Liu DW, Yan X, Jiang L, Yang ZY, Chu JP (2019). Comparative analysis of the diffusion kurtosis imaging and diffusion tensor imaging in grading gliomas, predicting tumour cell proliferation and IDH-1 gene mutation status. J Neuro-Oncol.

