# Supplementary material for: The diagnostic role of diffusional kurtosis imaging in glioma grading and differentiation of gliomas from other intra-axial brain tumours: a systematic review with critical appraisal and meta-analysis
Source: Neuroradiology. 2020 May 4;62(7):791–802. doi: 10.1007/s00234-020-02425-9 (PMC7311378; doi:10.1007/s00234-020-02425-9)
Supplement: Supplementary file 2 — (PDF 493 kb) [file 234_2020_2425_MOESM2_ESM.pdf]

# Search Strategy

- Date: 12 July 2018
- Research question: What is the diagnostic accuracy of MRI diffusional kurtosis in 1) differentiating high and low grade gliomas and 2) differentiating gliomas from other primary or secondary brain tumours
- Researchers:
- Librarian: Kate Brunskill (Deputy Librarian, Queen Square Library & Archive)
- Databases:
    - Pubmed
    - Medline via ovid
    - Embase
    - Scopus
- No language restrictions
- Number of articles:
    - Before checking for duplicates: 216
    - After removing of duplicates: 88
- Comments:

1) PUBMED:

Last search was done on: 12 July 2018

Search syntax:

("Glioma"[Mesh] OR "Brain Neoplasms"[Mesh] OR glioma[TW] OR gliomas[TW] OR (brain[TW] AND neoplasm*[TW])) AND ((diffusion[TW] AND kurtosis[TW]) OR (diffusional[tw] AND kurtosis[TW]) OR DKI[TW] OR “non Gaussian”[TW])

Number of returned articles: 50

---

2) Medline via ovid:

Last search was done on: 12 July 2018

Search terminology:

1. diffusional kurtosis.mp.
2. diffusion kurtosis.mp.
3. DKI.mp.
4. non gaussian.mp.
5. 1 or 2 or 3 or 4
6. exp Glioma/

7. exp Brain Neoplasms/
8. (glioma or gliomas or brain neoplasm*).mp. [mp=title, abstract, original title, name of substance word, subject heading word, keyword heading word, protocol supplementary concept word, rare disease supplementary concept word, unique identifier, synonyms]
9. 6 or 7 or 8
10. 5 and 9

Number of returned articles: 40

---

3) Embase

Last search was done on: 12 July 2018

Search engine is same as medline via ovid

1. diffusional kurtosis.mp.
2. diffusion kurtosis.mp.
3. DKI.mp.
4. non gaussian.mp.
5. 1 or 2 or 3 or 4
6. exp Glioma/
7. exp Brain Neoplasms/
8. (glioma or gliomas or brain neoplasm*).mp. [mp=title, abstract, original title, name of substance word, subject heading word, keyword heading word, protocol supplementary concept word, rare disease supplementary concept word, unique identifier, synonyms]
9. 6 or 7 or 8
10. 5 and 9

Number of returned articles: 69

---

4) Scopus

Last search was done on: 12 July 2018

Search syntax: TITLE-ABS-KEY ( *glioma* OR *gliomas* OR *"brain Neoplasm"* OR *"brain neoplams"* ) AND

TITLE-ABS-KEY ( ( *diffusion* AND *kurtosis* ) OR ( *diffusional* AND *kurtosis* ) OR *dki* OR *"non Gaussian"* OR *"non-Gaussian"* )

Number of returned articles: 57
