# Supplementary figures and images for: The diagnostic role of diffusional kurtosis imaging in glioma grading and differentiation of gliomas from other intra-axial brain tumours: a systematic review with critical appraisal and meta-analysis
Source: Neuroradiology. 2020 May 4;62(7):791–802. doi: 10.1007/s00234-020-02425-9 (PMC7311378; doi:10.1007/s00234-020-02425-9)

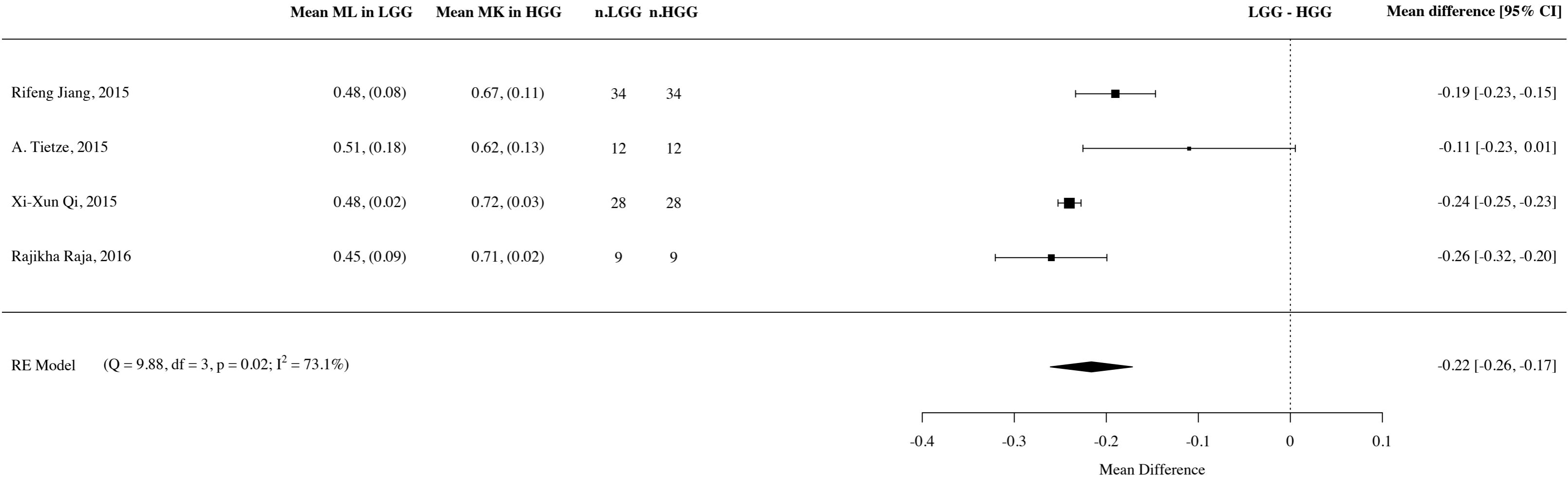

Supplement: Supplementary file 3 — (PDF 255 kb) [file 234_2020_2425_MOESM3_ESM.pdf]

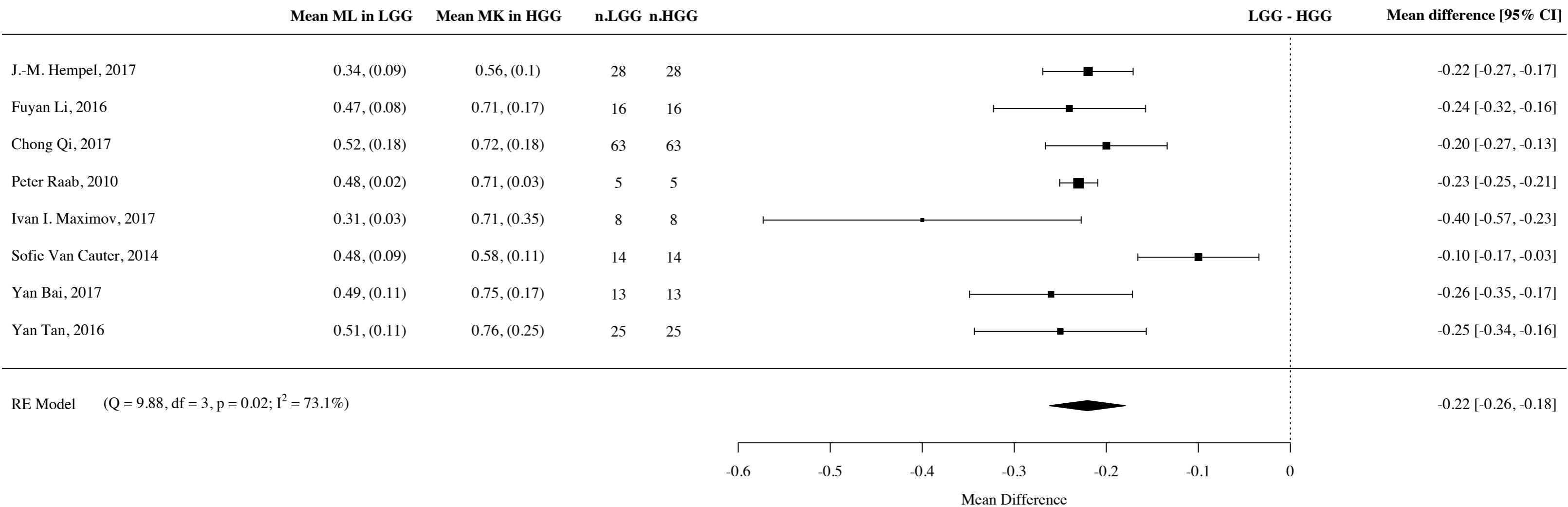

Supplement: Supplementary file 4 — (PDF 384 kb) [file 234_2020_2425_MOESM4_ESM.pdf]

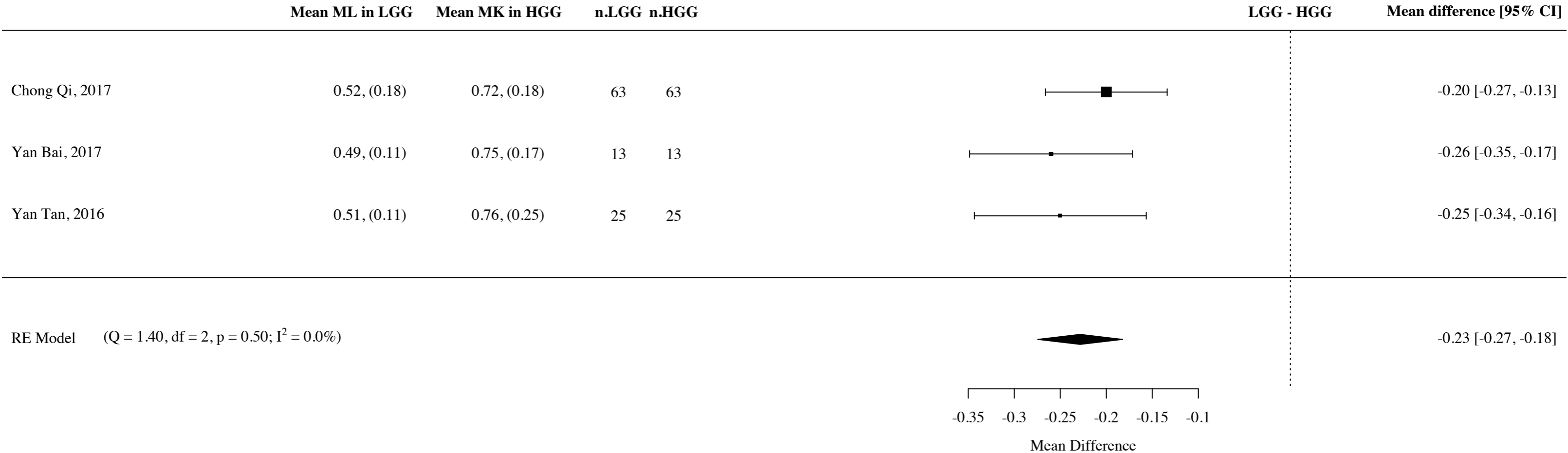

Supplement: Supplementary file 5 — (PDF 227 kb) [file 234_2020_2425_MOESM5_ESM.pdf]

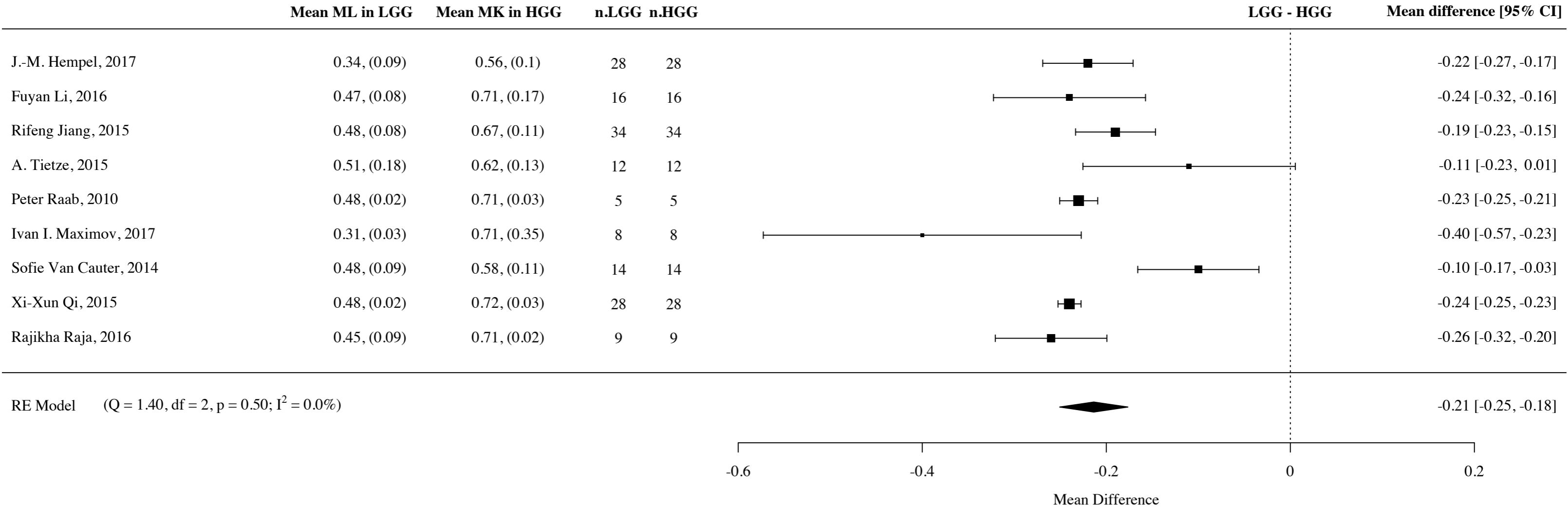

Supplement: Supplementary file 6 — (PDF 405 kb) [file 234_2020_2425_MOESM6_ESM.pdf]

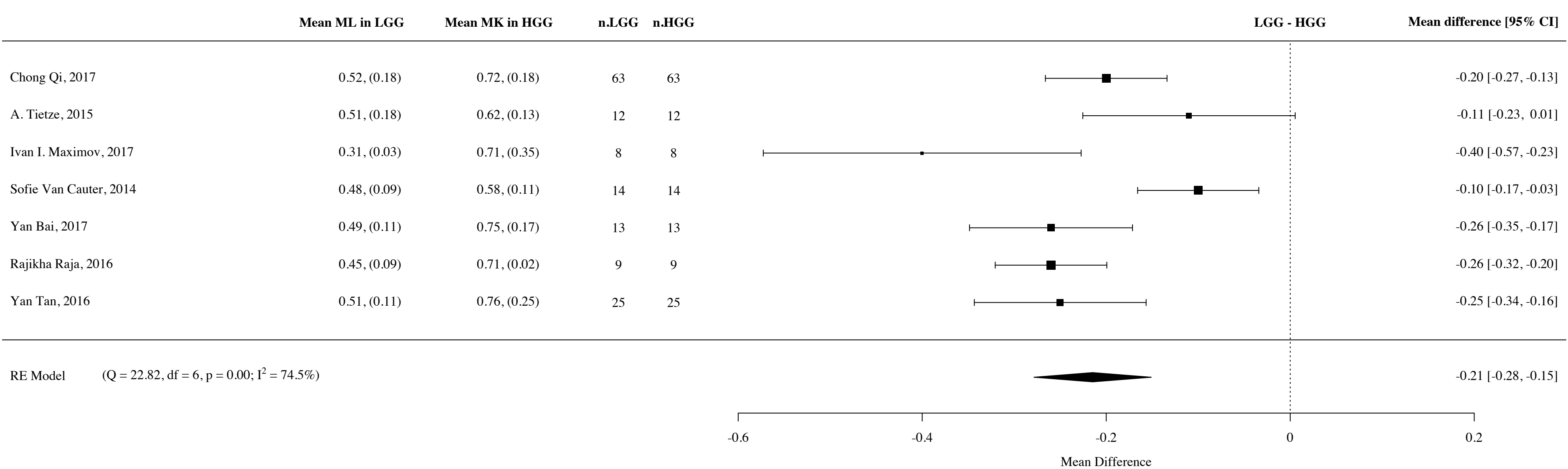

Supplement: Supplementary file 7 — (PDF 344 kb) [file 234_2020_2425_MOESM7_ESM.pdf]

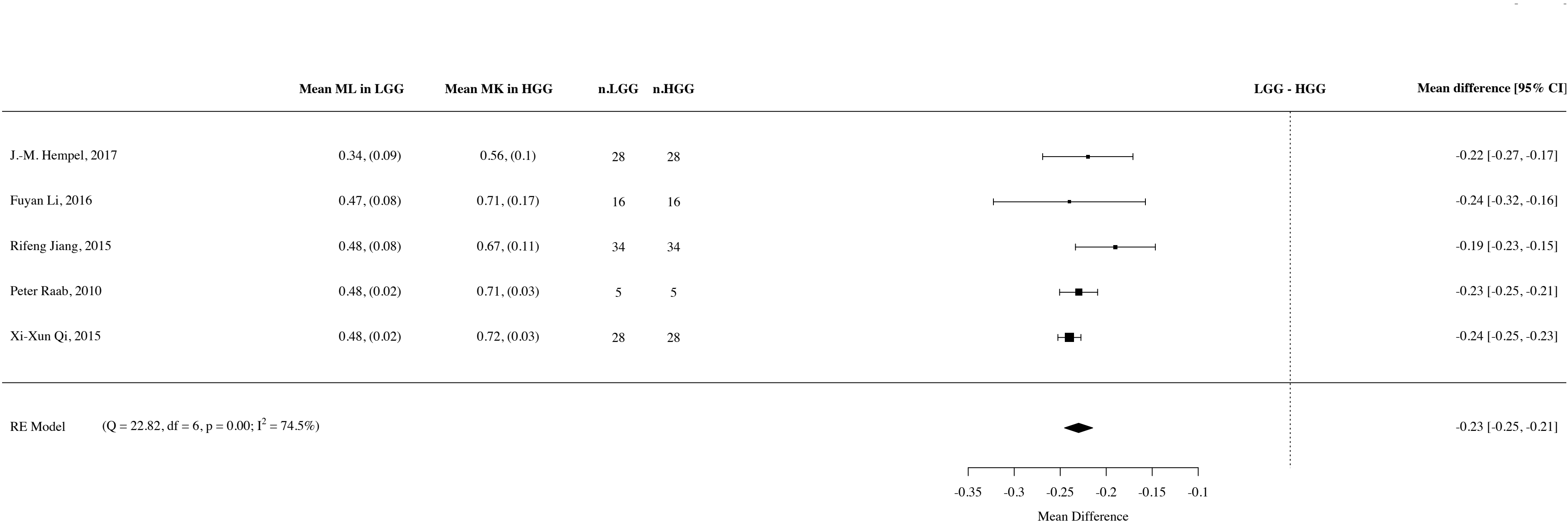

Supplement: Supplementary file 8 — (PDF 324 kb) [file 234_2020_2425_MOESM8_ESM.pdf]

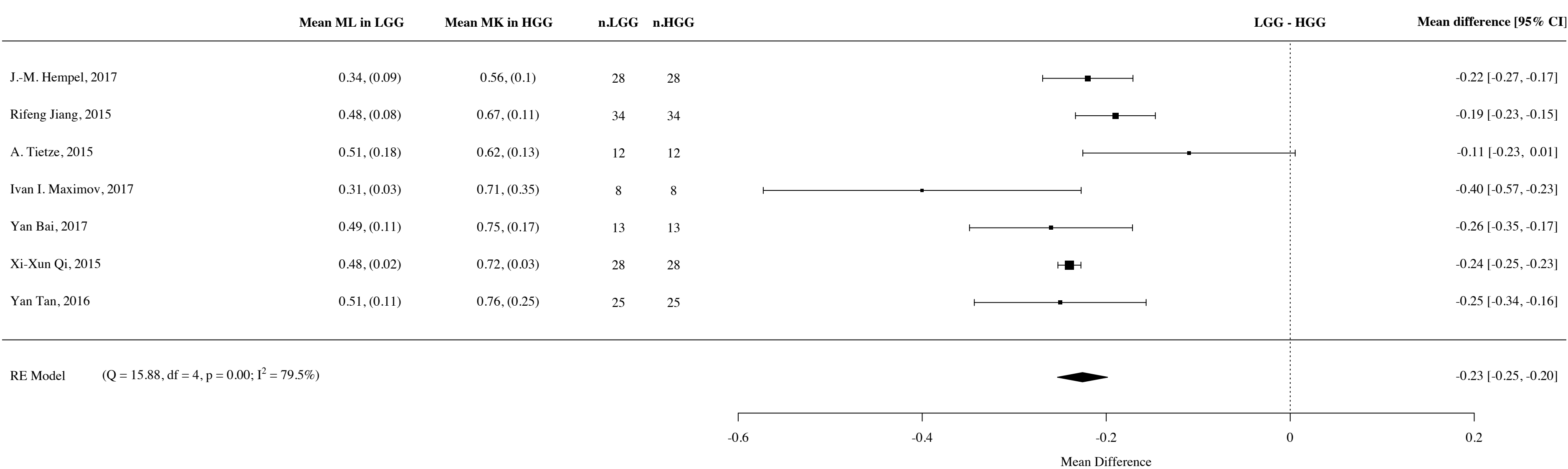

Supplement: Supplementary file 10 — (PDF 344 kb) [file 234_2020_2425_MOESM10_ESM.pdf]
