# Supplementary material for: The diagnostic role of diffusional kurtosis imaging in glioma grading and differentiation of gliomas from other intra-axial brain tumours: a systematic review with critical appraisal and meta-analysis
Source: Neuroradiology. 2020 May 4;62(7):791–802. doi: 10.1007/s00234-020-02425-9 (PMC7311378; doi:10.1007/s00234-020-02425-9)

	Mean ML in LGG	Mean MK in HGG	n.LGG	n.HGG	Mean difference [95% CI]
Fuyan Li, 2016	0.47, (0.08)	0.71, (0.17)	16	16	-0.24 [-0.32, -0.16]
Chong Qi, 2017	0.52, (0.18)	0.72, (0.18)	63	63	-0.20 [-0.27, -0.13]
Peter Raab, 2010	0.48, (0.02)	0.71, (0.03)	5	5	-0.23 [-0.25, -0.21]
Sofie Van Cauter, 2014	0.48, (0.09)	0.58, (0.11)	14	14	-0.10 [-0.17, -0.03]
Rajikha Raja, 2016	0.45, (0.09)	0.71, (0.02)	9	9	-0.26 [-0.32, -0.20]
RE Model (Q = 15.88, df = 4, p = 0.00; I^2 = 79.5%)					-0.21 [-0.26, -0.15]

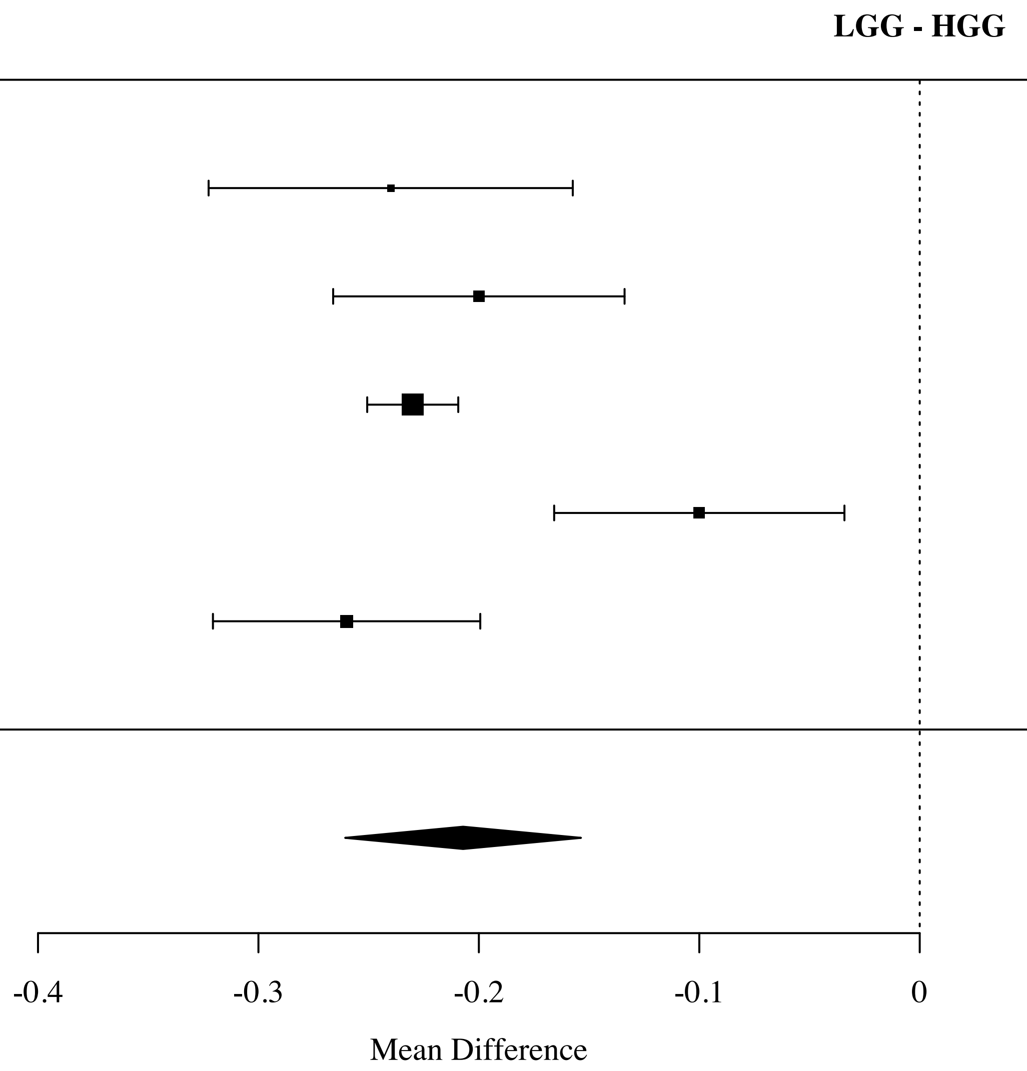

Supplement: Supplementary file 9 — (PDF 282 kb) [file 234_2020_2425_MOESM9_ESM.pdf]
